# Pyruvate as a Potential Beneficial Anion in Resuscitation Fluids

**DOI:** 10.3389/fmed.2022.905978

**Published:** 2022-07-12

**Authors:** Fang-Qiang Zhou

**Affiliations:** Fresenius Medical Care, Dialysis Center in Chicago, Chicago, IL, United States

**Keywords:** fluid therapy, resuscitation, hypoxia, metabolic acidosis, oral rehydration solution, pyruvate

## Abstract

There have been ongoing debates about resuscitation fluids because each of the current fluids has its own disadvantages. The debates essentially reflect an embarrassing clinical *status quo* that all fluids are not quite ideal in most clinical settings. Therefore, a novel fluid that overcomes the limitations of most fluids is necessary for most patients, particularly diabetic and older patients. Pyruvate is a natural potent antioxidant/nitrosative and anti-inflammatory agent. Exogenous pyruvate as an alkalizer can increase cellular hypoxia and anoxia tolerance with the preservation of classic glycolytic pathways and the reactivation of pyruvate dehydrogenase activity to promote oxidative metabolism and reverse the Warburg effect, robustly preventing and treating hypoxic lactic acidosis, which is one of the fatal complications in critically ill patients. In animal studies and clinical reports, pyruvate has been shown to play a protective role in multi-organ functions, especially the heart, brain, kidney, and intestine, demonstrating a great potential to improve patient survival. Pyruvate-enriched fluids including crystalloids and colloids and oral rehydration solution (ORS) may be ideal due to the unique beneficial properties of pyruvate relative to anions in contemporary existing fluids, such as acetate, bicarbonate, chloride, citrate, lactate, and even malate. Preclinical studies have demonstrated that pyruvate-enriched saline is superior to 0.9% sodium chloride. Moreover, pyruvate-enriched Ringer’s solution is advantageous over lactated Ringer’s solution. Furthermore, pyruvate as a carrier in colloids, such as hydroxyethyl starch 130/0.4, is more beneficial than its commercial counterparts. Similarly, pyruvate-enriched ORS is more favorable than WHO-ORS in organ protection and shock resuscitation. It is critical that pay attention first to improving abnormal saline with pyruvate for ICU patients. Many clinical trials with a high dose of intravenous or oral pyruvate were conducted over the past half century, and results indicated its effectiveness and safety in humans. The long-term instability of pyruvate aqueous solutions and para-pyruvate cytotoxicity is not a barrier to the pharmaceutical manufacturing of pyruvate-enriched fluids for ICU patients. Clinical trials with sodium pyruvate-enriched solutions are urgently warranted.

## Introduction

Fluid therapy is the first and essential treatment in perioperative and critical care patients. However, the selection of crystalloids and/or colloids, which depends on the pathophysiologic mechanism of various diseases; the fluid composition, property, and availability; and even clinicians’ individual preference, remains greatly debatable as each anion, such as acetate, bicarbonate, chloride, citrate, and lactate, in commercial fluid products has its own limitations in the resuscitation of critical care patients, generally contributing to iatrogenic resuscitation injury. In the past 3 decades, it has been well established that pyruvate, a weak acidic anion and the core element of glucose metabolism, holds unique beneficial physiological and pharmacological properties superior to those of the above-mentioned anions in current commercial fluids including intravenous (IV) crystalloids and colloids, as well as oral rehydration solution/salt (ORS). This review focuses on the essence of current fluid debates and the advantages, necessity, and potential clinical uses of sodium pyruvate-enriched fluids.

## Non-Optimality of Current Fluids

Normal saline (NS, 0.9% sodium chloride with equal sodium and chloride, 154 mmol/L) has been the most popular fluid in clinical practice for approximately 200 years. High-chloride saline including NS and hypertonic saline has a fatal limitation in that it induces iatrogenic hyperchloremia (1, 2), which leads to metabolic acidosis and kidney dysfunction mainly due to hyperchloremic renal vasocontraction and decline in the glomerular filtration rate (3, 4). In healthy volunteers, infusion of NS at 2 L/h results in hyperchloremia and a decrease in renal blood flow velocity and cortical tissue perfusion but does not cause any damage (5). However, hyperchloremia may exacerbate acid–base disorders and organ dysfunction in intensive care unit (ICU) patients, and hyperchloremia at hospital discharge may still be associated with the risk of 1-year patient mortality (6). Therefore, NS is neither optimal nor suitable for perioperative and ICU patients, but it remains the first choice for the treatment of hypochloremic alkalosis (1). A consensus states that NS is neither a normal nor physiological but an abnormal fluid, which should be replaced by balanced fluids in most patients, if possible.

To overcome the limitations of NS, Ringer’s and lactated Ringer’s solutions were produced around a century ago. Previous evidence shows that balanced fluid (lactated/acetated Ringer’s solution (LR/AR) or acetate-based Plasma-Lytes) is advantageous over NS in critically ill adults (7, 8), specifically in patients with diabetic ketoacidosis (9). However, recent findings with a large sample of patients reveal that balanced crystalloids (LR and AR) do not have advantages over NS regarding hospital-free days in non-critically ill patients (10) and that balanced fluids show no significant superiority over NS in reducing 90-day mortality in critically ill patients and kidney transplant graft function (11, 12). To date, the clinical outcomes of various fluids are still controversial in this respect, and one of the beneficial effects of current fluids may depend on specific subgroups of patient populations.

It has long been known that the serum lactate level is negatively associated with the reversibility of shock and is an independent risk factor of patients’ mortality in ICUs (13, 14). Notably, relative hyperlactatemia within the normal range is also independently associated with mortality in ICU patients (15). Regarding LR, there is a rise of 0.93 mmol/L in the mean serum lactate level in healthy volunteers after a bolus of 30 ml/kg (16). A large LR infusion likely exacerbates lactate accumulation in the resuscitation of patients with severe or decompensated shock, which may interfere with the diagnosis and treatment of lactic acidosis. Thus, LR is not optimally worthy of recommendation in critically ill patients (17). However, it is still controversial whether LR worsens lactic acidosis in ICU patients, including those with septic shock. Although LR generally does not decrease the lactate clearance, the persistence of hyperlactatemia during the first 24 h with a high L/P (lactate/pyruvate) ratio is still associated with a risk of multi-organ failure and death in clinical septic shock (18). The recommended solution is rather the acetate-based Plasma-Lyte solution (17, 19). Nevertheless, in a case report, an AR infusion of sufficient quantity induced lactic acidosis but did not cause any adverse effects (20).

Evidently, there is no consensus on the type of IV fluid, either crystalloids (balanced or non-balanced) or colloids (hydroxyethyl starch: HES 130/0.4, albumin, or plasma with various crystalloids as carriers), that is the best for most patients (21), as each fluid has advantages and disadvantages in the majority of patients.

On the other hand, the volume and speed of fluid delivery are also a critical concern in various clinical settings. Individualized goal-directed therapy (IGDT) is currently optimal in perioperative fluid management and critical care patients, but it still faces challenges. IGDT did not improve early renal function in a recent renal transplant study with a porcine model (22). Although very little or excessive fluid infusion can induce an immediate hemodynamic compromise and cause organ dysfunction (22), a recent study with a large sample of patients found that infusing at a slower (333 ml/h) vs. faster (999 ml/h) rate did not result in a statistical difference in 90-day mortality among ICU patients who were randomized in two groups to receive balanced solutions and NS, respectively (23).

Generally, although current fluids play an important role in healthcare, the ongoing debates about resuscitation fluids essentially reflect a crucial embarrassing clinical *status quo* that all current fluid products are not quite satisfactory in most clinical settings as an ideal selection, especially in ICU patients with severe and complex comorbidities. Therefore, a novel optimal fluid that overcomes most of the limitations of current fluids is warranted for most patients in a wide variety of severe clinical scenarios, particularly diabetic and elderly patients with or without organ comorbidities.

## Advantages of Pyruvate in Future Fluids

**FIGURE 1:**
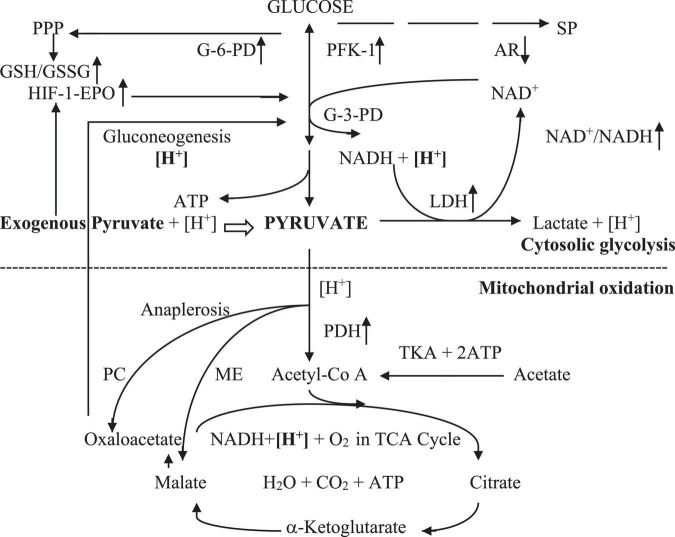
Exogenous pyruvate metabolism in cellular hypoxia. Exogenous pyruvate enters cell plasma with [H^+^]. The pyruvate or glycolytic pyruvate with [H^+^] spontaneously reduces to lactate with LDH free of energy in anoxia, leading to [**H**^ +^] consumption and increment of the NAD^+^/NADH ratio that promotes the glycolytic pathway at glyceraldehyde-3-phosphate dehydrogenase. Exogenous pyruvate also facilitates glycolysis by stimulating the HIF-1α-EPO signal pathway, increasing G-6PD activity, thereby preserving the PPP pathway and GSH/GSSG ratio. It inhibits AR activity in the sorbitol pathway likely by competing inhibition, enhancing NAD^+^/NADH also in the second step of sorbitol pathway. Thus, pyruvate sustains canonical glycolytic pathways and glycolytic ATP. It also inhibits LDH-A to decline the pyruvate reduction to lactate. Pyruvate enters mitochondria with [H^+^] and oxidates in hypoxia and normoxia by renovating inhibited PDH in the TCA cycle, resulting in mitochondrial ATP generation and [**H**^ +^] consumption. Also, it promotes the TCA cycle via anaplerosis with preservation of PC and ME activities. Hence, pyruvate reverses the Warburg effect. Pyruvate-based gluconeogenesis consumes additional [**H**^ +^] in relation to lactate-based one in cytosol. Pyruvate has the most powerful energetics with the least oxygen consumption in equal molar ATP generation among lactate, acetate, citrate, and malate oxidation. AR, aldose reductase; ATP, adenosine triphosphate; G-3-PD, glyceraldehyde-3-phosphate dehydrogenase; G-6-PD, glucose-6-phosphate dehydrogenase; GSH/GSSG, glutathione (reduced/oxidized); HIF-1-EPO, hypoxia-inducible factor-1-erythropoienin; [H^+^], hydrogen in cellular hydrogen pool; [**H**^+^], hydrogen consumed in a molar basis; LDH, lactate dehydrogenase; ME, malic enzyme; NADH/NAD^+^, nicotinamide adenine dinucleotide (reduced/oxidized); PC, pyruvate carboxylase; PDH, pyruvate dehydrogenase; PFK-1, phosphofructokinase-1; PPP, pentose phosphate pathway; SP, sorbitol pathway; TCA cycle, tricarboxylic acid cycle with oxidative phosphorylation; TKA, thiokinase.

Pyruvate is a key metabolite of glycolysis, which is reduced to lactate in anoxia or enters oxidative metabolism in the tricarboxylic acid (TCA) cycle in normoxia and hypoxia. The exogenous pyruvate metabolic profile in the intracellular environment can be simply illustrated, as shown in Figure 1.

Exogenous pyruvate in aqueous solutions is a unique anion that has pluripotent beneficial physiological and pharmacological properties to protect multi-organ structures and functions against various noxious insults, such as cardiogenic, hemorrhagic, traumatic, and septic shock. Specifically, pyruvate is a potent alkalizer used in preventing and treating hypoxic lactic acidosis, which is not only lethal but also lacks an ideal treatment agent in clinical practice (17). Recently, its beneficial properties over current anions in medical fluids, as mentioned before, are increasingly being recognized in fluid resuscitation, mainly due to the following beneficial bioactive characteristics.

### Increase in Cellular Hypoxia Tolerance

An experiment conducted in 2012 showed that pyruvate protects the function of red blood cells (RBCs), which are the most abundant tissues in humans and exclusively depend on anaerobic glycolysis to produce glycolytic ATP due to the absence of mitochondria (24). Moreover, the delayed decline in ATP levels and ATPase activities of dogs’ RBCs in the extracorporeal circuit primed with sodium pyruvate/chloride saline vs. sodium chloride saline demonstrates that pyruvate alone is beneficial for cell metabolism throughout the body, even in anoxia (24). This investigation validates two previous findings: (1) pyruvate preserved brain ATP levels and prolonged survival in rats subjected to anoxia by exploring a pure nitrogen atmosphere in the 1960s (25), and (2) pyruvate protected against anoxic injury in the anoxic perfusion of hepatocytes, as indicated by decreases in superoxide generation and lactate dehydrogenase (LDH) release, and against reoxygenation injury in rats in the 1990s (26). Recently, infusion of RBCs with pyruvate restoration from storage-induced damage robustly alleviated liver injury in rats, indicating the potential significance of pyruvate protection of RBCs in clinical resuscitation (27). Evidently, it also preserves ATP generation in hypoxia (28, 29). These findings substantiate a fundamental and core fact that exogenous pyruvate preserves canonical glycolysis and glycolytic ATP, which is essential for each cell in both hypoxia and anoxia (see below).

### Reactivation of Pyruvate Dehydrogenase and Reversal of Warburg Effect

Pyruvate stimulates pyruvate dehydrogenase (PDH) via the direct inhibition of PDH kinase (PDK), similar to dichloroacetate (DCA, a classic PDH stimulator) (30, 31), and probably via the enhancement of PDH phosphatase 2 (PDP2) expression (32). Recent findings further demonstrated that pyruvate preserved the nicotinamide adenine dinucleotide oxidative form/reduced form (NAD^+^/NADH) ratio, restored pyruvate kinase (PK), and retarded the sorbitol pathway by the competitive inhibition of aldose reductase, resulting in the promotion of classic glycolytic pathways (Figure 1). It also accelerated glucose oxidative phosphorylation by rejuvenating the suppressed PDH and pyruvate carboxylase (PC) activities in traumatic brain injured and hemorrhagic rats and diabetic *db/db* mice (31, 33). Alternatively, it can inhibit LDH-A activity, which is usually overexpressed in hypoxia, diabetes, and cancer, facilitating oxidative metabolism in cells (34–36).

Several pieces of preclinical evidence revealed that pyruvate protected from diabetic cataract and retinopathy and increased blood insulin levels (33, 37, 38). Preliminary case reports also confirmed the findings that pyruvate protected against diabetes by reducing daily insulin doses in type 1 diabetes (39, 40). Accordingly, it can reverse glucometabolic disorders in diabetes and trauma and hemorrhage, turning the vicious circle of diabetic glucometabolic disturbance into a virtuous circle (33) as it reversed the Warburg effect by DCA in cancer.

### Correction of Severe Metabolic Acidosis

Pyruvate is a potent alkalizer via the rapid metabolic consumption of hydrogen ions (proton, [H^+^]) through the LDH reduction reaction, which is a systemic alkalizing enzymatic reaction, coupled with an increase in the NAD^+^/NADH ratio, and the gluconeogenesis pathway in the cytosol in addition to oxidative phosphorylation in the mitochondria (Figure 1). Although it is a weaker acidic anion of sodium salt with a low buffer capacity of *p*Ka 2.49, pyruvate favors a rise in blood plasma pH accordingly (17, 41). Pyruvate has the potential to effectively correct hypoxic lactic acidosis in critically ill patients, as repeatedly demonstrated with IV or oral pyruvate in small or large animal studies, which resulted in approximately doubled survival (42–45). A case report described the effectiveness of a high dose of oral sodium pyruvate in robustly attenuating continuous severe lactic acidosis in a child with Leigh syndrome due to a novel mutation in the *PDH E1*α gene (46). Another study involving 11 adult patients with the mitochondrial disease also revealed significant decreases in plasma and lateral ventricular lactate and the L/P ratio accompanied by clinical improvements as a result of pyruvate therapy (47). Therefore, pyruvate enriched IV and oral solutions are excellent in preventing and treating severe metabolic lactic acidosis in various severe clinical scenarios, although no data from clinical resuscitation have yet been reported.

### Inhibition of Oxidative/Nitrosative Stress and Inflammation

Endogenous pyruvate is a natural and potent antioxidant agent. It is widely known that it can effectively exert a dual effect of antioxidant/nitrosative stress by directly interacting with reactive oxygen/nitrogen species *via* a non-enzymatic stoichiometric reaction and indirect action with redox potentials, mainly NAD^+^/NADH and GSH/GSSG (glutathione-reduced form/oxidative form) (48). A recent comprehensive review clearly illustrated the critical significance of reducing equivalents, including NAD^+^ and GSH, in maintaining cellular redox homeostasis and modulating cellular metabolism (49). There are numerous pieces of evidence that show pyruvate robustly increases both NAD^+^/NADH and GSH/GSSG ratios in tissues in various injuries (33, 42, 50). In addition, it inhibits inflammatory reactions, including both infiltration of inflammatory cells and secretion of inflammatory mediators, such as cytokines including IL-2 and IL-6, TNF-α, and high-mobility group box-1 (HMGB-1) (43).

### Stimulation of HIF-1 and Protection of the Mitochondria

As previously discovered, pyruvate can directly stimulate the hypoxia-inducible factor-1α-erythropoietin (HIF-1α-EPO) signal pathway by inhibiting the HIF-prolyl hydroxylase domain (PHD) to avoid HIF-1α proteasomal degradation in both hypoxia and normoxia and the subsequent elevation of the gene expression and content of EPO (51, 52). The activation of the HIF-1α-EPO pathway further stimulates the downstream enzymes to improve energy metabolism and mitochondrial energetics. On the other hand, it also protects the mitochondrial structure and endoplasmic reticulum function and protects against cellular apoptosis in various insults (53–55). Therefore, the effects of pyruvate can continue for at least several hours after the rapid decline in the peak plasma level to normal in approximately 1 h following the termination of pyruvate infusion (51). In addition, pyruvate can inhibit the formation and deposition of advanced glycation end products (AGEs), which are one of the major pathogenic triggers in organ complications, in hypoxia and diabetes as an AGE antagonist (33, 56).

### Stimulation of Insulin Secretion

Sodium pyruvate like methyl pyruvate works as an insulin stimulator. Pyruvate metabolism in the mitochondria is intimately involved in glucose-stimulated insulin secretion (GSIS) and the PC activity, on which pyruvate oxidative metabolism partially depends. PC facilitates the anaplerotic flux, which also plays a critical role in insulin secretion from pancreatic islets (57, 58). It can effectively control diabetes progression and organ complications with restored insulin levels in *db/db* mice (33). Therefore, exogenous pyruvate enhances insulin secretion in islet β-cells even in type 1 diabetic patients to the extent of hypoglycemia (39, 40). Intriguingly, in children with citrin deficiency, oral pyruvate induces significant enhancements of fasting insulin levels for months (59). Furthermore, in rats subjected to severe scald with multiple organ dysfunction syndrome, direct peritoneal resuscitation with pyruvate-based peritoneal dialysis solution (P-PDS) containing glucose still effectively protects the function of the islet β-cells, as demonstrated by a higher homeostasis model assessment of β-cell (HOMA-β) level without hyperglycemia (60). Thus, pyruvate is beneficial in the resuscitation of critical care patients, specifically those with diabetes and elders (61).

### Exertion of Anti-aging

The decrease in NAD^+^ cellular levels and the increase in senescence cells (a permanent cell cycle arrest but living cell) in tissues are closely associated with natural aging (62, 63). Pyruvate can spontaneously generate NAD^+^, mainly through the LDH reduction reaction coupled with NADH oxidation free of oxygen and energy, enhancing NAD^+^ levels in the cytosol on an equimolar basis. Alternatively, the clearance of senescence cells by a senolytic improved multi-organ (kidney and heart) functions and extended the healthy lifespan in mice. A pilot study in patients with idiopathic pulmonary fibrosis first showed promising clinical improvements with oral senolytics (64). Importantly, a cellular study found that pyruvate prevented cellular senescence in normal human fibroblasts by increasing NAD^+^ generation *in vitro* and mimicking human skin *in vivo* (53). Notably, it also showed the protection of DNA repair in rodents and human cells (65). Therefore, pyruvate acts generally as both a novel NAD^+^ substitute and a new senolytic substance, although further exploration and demonstrable data are warranted (66).

The unique properties of pyruvate described above are superior to those of the anions currently found in fluids used for ICU patients, which lack the properties of pyruvate or have much inferior ones; even malate shows the capacity to effectively eliminate hypoxic lactic acidosis in sufficient amounts cannot be metabolized in anoxia (67), leading to no protection of RBCs. It also possesses the lowest oxygen consumption rate per ATP generation among acetate, citrate, lactate, and malate (17). Therefore, pyruvate is more metabolically protective, especially regarding glucometabolic and acid–base balance and multi-organ function, predominantly in the heart, brain, liver, kidney, and intestine, than current anions in various pathogenic attacks. Alternatively, hypoxia, glucometabolic disturbance, metabolic acidosis, oxidative stress and inflammation, mitochondrial dysfunction, and cellular apoptosis, against which pyruvate protects in multiple organs, are all pathophysiological processes that are shared in most critical illnesses. Accordingly, the favorable pleiotropic bioactivities of pyruvate effectively meet the clinical needs and are beneficial in most patients with or without parenchymatous organ comorbidities.

## Pyruvate Saline Superior to Normal Saline

Intriguingly and significantly, pyruvate saline, sodium pyruvate 50 mM plus sodium chloride 104 mM, is advantageous over 0.9% sodium chloride (NS, 154 mM) in preserving ATP generation and ATPase activity in the RBCs of dogs simulated bypass surgical procedures *in vitro* (24). The ATP product from RBCs is glycolytic ATP, an essential component of ATPase in all cells in the body for several basic cellular functions, such as ion polarization of plasma membranes and maintenance of membrane integrity by Na^+^-K^+^-ATPase, and organelle pH regulation by vacuolar ATPase (V-ATPase). The data also demonstrate the inhibition of inflammatory reactions of RBCs by pyruvate, as shown by the reduction of endothelial nitric oxide synthase (eNOS) and nitric oxide (NO) in plasma (24). RBCs as an oxygen sensor that triggers the dilation of the microvascular circulation by releasing glycolytic ATP in addition to oxygen delivery are intimately associated with oxygen supply in tissues (68). As aforementioned, RBCs rejuvenated by pyruvate could attenuate liver injury after the blood infusion, indicating the improvement of tissue hypoxia in rats (27). In this respect, its protective effects on stored RBCs were also displayed early in rat models of renal oxygenation and in clinical bypass surgery studies (24). Furthermore, either a high or regular (28 mM) pyruvate concentration preserved the partial pressure of arterial oxygen (*p*aO_2_) and systemic and cerebral oxygen delivery and consumption in shock IV resuscitation, further demonstrating the attenuation of tissue hypoxia by pyruvate (42, 44). Oral pyruvate also resulted in the preservation of *p*aO_2_ in severe shock rehydration (69). Additionally, preliminary data showed that it might also improve the RBC oxygen–hemoglobin dissociation curve against peroxide stress (70). The pyruvate effect on *p*aO_2_ in shock resuscitation should be specifically and intensively investigated.

Furthermore, IV pyruvate saline protection of the kidney was first preliminarily reported in China a decade ago in rats subjected to burn shock with 50% TBSA III (total body surface area, full-thickness scald), compared to an equal volume of NS infusion, although pyruvate renoprotection had been earlier investigated (71, 72); the kidney vascular permeability, tissue water content, hematocrit, and serum creatinine levels 4 h after the scald were significantly increased in the NS group compared to the pyruvate group, while no significant difference was found between the pyruvate group and the sham group (72). Although the histopathological alteration was not investigated, the results apparently revealed that pyruvate might not only prevent kidney injury generally induced by NS due to hyperchloremia in severe shock resuscitation, but also protect the kidney function from burn shock. Its renoprotection was further appreciated afterward, including the protection against diabetic nephropathy (33, 73). In addition, pyruvate also protects systemic endothelial cells in addition to RBCs and neutrophils against oxidative stress (74). These findings provide a convincing basis for using pyruvate saline, rather than NS in ICU patients in future clinical practice, although further intensive studies are required. Therefore, the predictable advantages of pyruvate saline are that it prevents iatrogenic hyperchloremia and protects multi-organ function as a therapeutic agent of organ metabolic and functional aberrances and a volume expander in fluid resuscitation. In these terms, 1.7% sodium pyruvate (154 mM) also showed more promising results than 0.9% sodium chloride by 90 vs. 30% survival at 90 min after fluid infusion during resuscitation for a severe hemorrhagic shock in a rodent model (75).

On the other hand, studies have demonstrated the advantages of PR over LR in shock resuscitation, particularly in effectively correcting hypoxic lactic acidosis, inhibiting apoptosis, and prolonging survival in animal studies (43, 44, 76).

It is worth noting that the regular pyruvate concentration (28 mM) with a low dose was as efficient as high doses for metabolic improvement and multi-organ protection in early reports (42, 43).

Importantly, a very small amount of pyruvate in cardioplegia showed apparent cardio-protection in clinical bypass surgery (77). In parallel, pyruvate-enriched ORS (Pyr-ORS; 0.35%: equimolar pyruvate replacement of bicarbonate or citrate in WHO-ORS I, II, and III) is superior to the latter three ORSs in correcting lactic acidosis, protecting multiorgan, and increasing survival in the shock rehydration of rats and dogs (Table 1) (45, 51, 69, 78).

**TABLE 1:** Compositions of oral rehydration salt.

	Alkalizer (g/L)	NaCl (g/L)	KCl (g/L)	Glucose (g/L)	mOsm/L	Acidosis correction
WHO-ORS (I) Bicarbonate	2.5	3.5	1.5	20.0	331	No effect on hypoxic LA
WHO-ORS (II) Citrate	2.9	3.5	1.5	20.0	311	Ibid
WHO-ORS (III) Citrate	2.9	2.5	1.5	13.5	245	Ibid
Pyr-ORS Pyruvate	3.5	3.5	1.5	20.0	335	Hypoxic LA correction
(regular osmolarity)						
Pyr-ORS Pyruvate	3.5	2.0	1.5	13.5	247	Ibid
(low osmolarity)						

*WHO-ORS, World health organization-guided oral rehydration salt; Pyr-ORS, pyruvate-enriched oral rehydration salt; LA, lactic acidosis.*

Furthermore, early studies documented that pyruvate-based PDS (P-PDS) was much more effective than regular commercial lactate-based PDS (L-PDS) in improving human blood neutrophils’ intracellular pH and superoxide generation (79, 80). Regarding direct peritoneal resuscitation, recent animal studies also found that P-PDS was significantly advantageous over L-PDS in reversing visceral hypoperfusion, correcting metabolic acidosis, and protecting the intestinal barrier (81–83). Furthermore, as an example of colloids, pyruvate as a novel carrier in HES 130/0.4 significantly demonstrated kidney protection (as well as the intestinal barrier preservation: unpublished data) in the fluid resuscitation of rats subjected to lethal burn shock, compared to regular carriers in commercial HES 130/0.4 products (21).

All these indicate that pyruvate replacement of current anions, such as acetate, chloride, and lactate, in carrier solutions of colloids would robustly improve the clinical benefits of synthetic or natural colloids, although malate has not been compared till now.

It is rational and expected that on a monolithic view, pyruvate as a novel component in fluid therapy would greatly ameliorate the prognosis of diseases and clinical outcomes, particularly under IGDT, in perioperative fluid management and ICU patients, especially those with diabetes and elders, as the initial clinical indication. For example, clinical studies demonstrate that major abdominal surgery can induce a substantial PDH decrease with glucose metabolic dysregulation in muscles, probably due to an increase in PDK activity and insulin resistance (84, 85). Thus, pyruvate-enriched fluids would be an optimal selection to prevent postoperative hyperglycemia, probably due to its preservation of PDH and islet function (30–33, 60). At present, if pyruvate solutions were compassionately used in severe COVID-19 patients, clinical outcomes might have greatly improved (86, 87). In the future, pyruvate-enriched fluids may encompass other medical solutions specifically used for RBC storage, cell salvage, organ preservation, cardioplegia, peritoneal dialysis, priming fluid for cardiopulmonary bypass circuits, and others (24, 27, 77, 80, 88).

## Safety and Feasibility of Pyruvate Fluids

Sodium pyruvate has been intravenously infused in humans since the 1930s. One early study used IV 3.5 g% pyruvate at 10 mg/kg (20 ml/70 kg) for 1–1.5 min in 21 healthy subjects and 27 patients subjected to Vit B1 deficiency (89); another study used 18.8 g of 12% pyruvate in three non-psychotic and four schizophrenic patients (90). Then, 10 g of pyruvate (100 ml of 10% solution) was infused in 18 non-diabetic subjects and 19 diabetic patients to investigate the secretion of pyruvate in urine in 1960 (91). No unexpected effects were observed in all these subjects. Subsequently, many clinical reports with high pyruvate doses in product qualities at the time demonstrated its safety with the absence of adverse effects. In 1996, the first IV pyruvate treatment of chronic liver diseases was reported in 11 patients with pyruvate 54–86.4 g/d for 10 days, followed by a consecutive report; the results showed a promising improvement in liver functions and pathological alterations (92, 93). The first intracoronary pyruvate infusion (totally 1.53 g and 3.05 g in 30 min, according to the calculation) was studied in eight patients with dilated cardiomyopathy in the Lancet in 1999; the hemodynamic measurements, including increases in the cardiac index and stroke volume and a decrease in pulmonary capillary wedge pressure, demonstrated the clinical cardio-protection due to pyruvate with a favorable inotropic effect (94). Additional reports on severe heart failure with intracoronary pyruvate of approximately 6.0 g in 30 min and others with cardioplegia on small doses of pyruvate and several IV loading tests of 10.0 g/4 min or 0.5 g/kg in 10 min all further demonstrated its clinical effectiveness and safety (77, 95–97). The highest oral dose (initially 0.25 g/kg t.i.d. with a maintenance dose of 0.5 g/kg t.i.d.) in 11 patients subjected to mitochondriopathy for 11 months and the same dose in a case with Leigh syndrome for years showed clinical improvements without adverse effects (46, 47). The reported clinical side effects were local pain induced by IV pyruvate at high concentrations and gut irritation or dizziness due to oral pyruvate at high doses (39, 91). The only report presents the case of a child with restrictive cardiomyopathy who received pyruvate infusion and died shortly after the pyruvate loading test (98); however, the causation between pyruvate and death was not confirmed (99).

However, the U.S. FDA has not verified its clinical use to date. The FDA approved a pyruvate-based product (Rejuvesol) in the 1990s, which was the sole commercial pyruvate-compounded solution stored at 4°C for the rejuvenation of stored RBCs *in vitro* before infusion (100). Early data regarding its acute toxicology showed that oral pyruvate LD_50_ was over 10.0 g/kg in rats, and IV pyruvate LD_50_ was over 1.25 g/kg in mice; thus, pyruvate was considered non-toxic in humans (39, 92).

The most crucial issue is the instability of pyruvate aqueous solutions at room temperature (101). Several patents on pyruvate clinical uses including fluid therapy were filed or issued during the last decades (102, 103), strongly indicating that the pivotal role of pyruvate in clinical medicine has been well recognized. However, no pharmaceutical manufacturers can produce pyruvate aqueous solutions worldwide to date. Although pyruvate dimers, such as para-pyruvate, which are cytotoxic in cell experiments *in vitro* (104), are spontaneously generated in pyruvate aqueous solutions at room temperature, the appropriate acidic environment can inhibit the non-enzymatic aldol-type condensation reaction (101). A patent on the long-term stability of pyruvate aqueous solutions was issued a decade ago following the experimental data of over 99.0% of pyruvate in water with a pH of approximately 4.5 at 25°C for 2 years (US patent: 8,835,508 B2, 2014). To date, there have not been any data regarding para-pyruvate cytotoxicity in humans *in vivo*. The purity of sodium pyruvate powders was merely over 98.0%, which probably included approximately 1.0% para-pyruvate, several decades ago when high doses of the product were safely infused in several hundreds of patients and healthy adults without acute adverse effects, as mentioned before, indicating that the toxicity of pyruvate aqueous solutions in humans is not realistically true, which is inaccurately referred in many references of ethyl pyruvate (EP) studies (105). Accordingly, pyruvate saline and pyruvate Ringer’s solution with pH 4.5–5.0 would be a long-term stable solution at room temperature without cytotoxicity as 5% glucose-NS but with a similar acidic pH in the clinical scenario. Furthermore, the pyruvate raw product is cheaper, and thus there are many suppliers. It is highly possible and essential to consider pharmaceutically manufacturing pyruvate-enriched fluids with a long-term shelf life for clinical practices today. At least, an IV preparation of sodium pyruvate powder injection should first be feasibly considered for emergency or compassionate use (77, 86).

On the other hand, oral pyruvate (0.35%) in a modified Pyr-ORS formula, which increases intestinal mucosal blood flow, energy metabolism, and Na^+^-K^+^-ATPase activity, protects intestinal barrier structure and function and enhances sodium and water absorption, thus resulting in lactic acidosis correction, multi-organ protection, and survival prolongation, relative to WHO-ORS counterparts (45, 69, 78, 106). Pyr-ORS as a pyruvate-containing beverage (107), which is approved by the FDA as a dietary supplement, may be efficient in prehospital rescue to take advantage of the “golden window” in the rehydration of acutely injured patients, particularly in an ambulance or resource-poor settings, in a large scale such as during earthquakes and terrorist attacks, as pointed out previously (51, 78). Recently, an experienced pyruvate research team strongly recommended the clinical use of pyruvate again (108). Taken together, although prior case reports with pyruvate applications were not sufficiently of high quality and did not have large group sizes, the overall results strongly demonstrate an irrefutable fact that pyruvate is clinically promising relative to all the drawbacks of the major anions found in contemporary resuscitation solutions. Pyruvate-enriched fluids may be a preferred choice via IV, oral, or peritoneal administration in the future for clinical shock resuscitation and the first choice for hemorrhagic shock (109). ORS therapy has been demonstrated in a top hospital to be effective in the patients of the whole hospital including those in the emergency room (110); however, Pyr-ORS has been shown to be more effective than WHO-ORS in various illnesses. Nevertheless, there has been no clinical study yet about the effects of IV or oral pyruvate on shock resuscitation, but novel oral pyruvate combined with nicotinamide has demonstrated its effectiveness in improving retinal ganglion cell function in human glaucoma (111).

Based on the current understanding of fluid therapy in ICU patients, it is recommended to prescribe fluids based on the condition of each individual patient and to understand the benefits of each solution regarding an individual ICU patient, as both are crucial (112). In contrast to existing commercial fluids, novel pyruvate-enriched fluids would be both a volume expander and a therapeutic agent in fluid resuscitation and will be appealing to clinicians as they could simply select them in various clinical settings.

Finally, numerous animal studies demonstrated that EP (a lipophilic ester derivative of pyruvate) in fluid resuscitation, such as the Ringer’s ethyl pyruvate solution (REPS), functions as well as sodium pyruvate (105), which strongly supports the effects of sodium pyruvate in the novel fluids. However, EP cannot correct severe metabolic acidosis, even if hyperlactatemia is improved (113), probably due to its hydrolysis to the pyruvate moiety with [H^+^] production. It is worth noting that EP works in animals but not in humans (114), and it was failed in a phase II clinical trial in 2009 (115). Moreover, a recent doubt was also raised about its clinical use (116), whereas sodium pyruvate is demonstrably effective and safe in humans, as indicated in many clinical tests over the last half century.

## Conclusion

Pyruvate is a multifactorial beneficial anion superior to the current anions, such as acetate, bicarbonate, chloride, citrate, lactate, and others, found in crystalloids and colloid fluids used for resuscitation. Pyruvate can protect metabolic homeostasis and multi-organ function in varying injuries in addition to avoiding iatrogenic adverse effects, such as resuscitation injury, of current fluids used in clinical resuscitation. Regarding the overall concept, the clinical advent of pyruvate-enriched formulations introduces a novel generation of fluid therapy to overcome the limitations of current fluids and be the first preferable fluid employed in most patients. Pyruvate applications would end the fluid debate; profoundly improve prognostic outcomes in ICU patients, especially those with diabetes and older patients; shorten the hospital stay; and enhance the quality of social healthcare. Clinicians and drug manufacturers should recognize that the long-term instability of pyruvate aqueous solutions and para-pyruvate cytotoxicity *in vitro* are almost not limitations or barriers to the pharmaceutical manufacturing of pyruvate-enriched fluids for clinical use. Randomized clinical trials of pyruvate in fluid resuscitation are urgently warranted.

## Author’s Note

The author is a retired independent medical researcher. The opinions or assertions contained herein are not a reflection of the view of Fresenius Medical Care, Dialysis Centers in Chicago, IL, United States.

## Data Availability Statement

The original contributions presented in the study are included in the article/supplementary material, further inquiries can be directed to the corresponding author/s.

## Author Contributions

FQZ prepared the entire manuscript draft and finally completed and revised the current version of the manuscript.

## Conflict of Interest

The author declares that the research was conducted in the absence of any commercial or financial relationships that could be construed as a potential conflict of interest.

## Publisher’s Note

All claims expressed in this article are solely those of the authors and do not necessarily represent those of their affiliated organizations, or those of the publisher, the editors and the reviewers. Any product that may be evaluated in this article, or claim that may be made by its manufacturer, is not guaranteed or endorsed by the publisher.

## Abbreviations

AGEs: advanced glycation end products
ATP: adenosine triphosphate
AR: acetated Ringer’s solution
DCA: dichloroacetate
EP: ethyl pyruvate
GSH/GSSG: glutathione reduced form/oxidative form
HES: hydroxyethyl starch
HIF-1 α -EPO: hypoxia-inducible factor-1 α -erythropoietin
[H^+^], hydrogen: proton
ICU: intensive care unit
IGDT: individualized goal-directed therapy
IV: intravenous
LDH: lactate dehydrogenase
L/P: lactate/pyruvate ratio
L-PDS: lactate-based peritoneal dialysis solution
LR: lactated Ringer’s solution
NAD^+^/NADH: nicotinamide adenine dinucleotide oxidative form/reduced form
NS: normal saline
ORS: oral rehydration solution/salt
*p*aO2: partial pressure of arterial oxygen
PDH: pyruvate dehydrogenase
PDK: pyruvate dehydrogenase kinas
PDP2: pyruvate dehydrogenase phosphatase 2
PC: pyruvate carboxylase
PHD: prolyl hydroxylase domain
PK: pyruvate kinase
P-PDS: pyruvate-based PDS
RBCs: red blood cells
TCA: tricarboxylic acid cycle.
